# Immune profiling of mouse lung adenocarcinoma paraffin tissues using multiplex immunofluorescence panel: a pilot study

**DOI:** 10.1186/s42826-024-00210-w

**Published:** 2024-06-14

**Authors:** Jie Zhai, Auriole Tamegnon, Mei Jiang, Renganayaki Krishna Pandurengan, Edwin Roger Parra

**Affiliations:** Department of Translational Molecular Pathology, Unit 951, The University of Texas MD Anderson Cancer Center, 2130 Holcombe Blvd, Houston, 77030 TX USA

**Keywords:** Tumor microenvironment, Spatial distribution, Multiplex immunofluorescence, Multispectral image analysis, Murine tumor

## Abstract

**Background:**

Immune profiling has become an important tool for identifying predictive, prognostic and response biomarkers for immune checkpoint inhibitors from tumor microenvironment (TME). We aimed to build a multiplex immunofluorescence (mIF) panel to apply to formalin-fixed and paraffin-embedded tissues in mice tumors and to explore the programmed cell death protein 1/ programmed cell death 1 ligand 1 (PD-1/PD-L1) axis.

**Results:**

An automated eight-color mIF panel was evaluated to study the TME using seven antibodies, including cytokeratin 19, CD3e, CD8a, CD4, PD-1, PD-L1, F4-80 and DAPI, then was applied in six mice lung adenocarcinoma samples. Cell phenotypes were quantified by software to explore the co-localization and spatial distribution between immune cells within the TME. This mice panel was successfully optimized and applied to a small cohort of mice lung adenocarcinoma cases. Image analysis showed a sparse degree of immune cell expression pattern in this cohort. From the spatial analysis we found that T cells and macrophages expressing PD-L1 were close to the malignant cells and other immune cells.

**Conclusions:**

Comprehensive immune profiling using mIF in translational studies improves our ability to correlate the PD-1/PD-L1 axis and spatial distribution of lymphocytes and macrophages in mouse lung cancer cells to provide new cues for immunotherapy, that can be translated to human tumors for cancer intervention.

**Supplementary Information:**

The online version contains supplementary material available at 10.1186/s42826-024-00210-w.

## Background

With the application and development of immunotherapy, cancer treatment has entered a new era in recent years. It was validated that immunotherapy is an effective treatment strategy in various types of cancer, such as non-small cell lung cancer (NSCLC), melanoma, and renal cell carcinoma [1–3]. Immune checkpoint inhibitors, essential components of immunotherapy, are antibodies targeting immune checkpoints like programmed cell death protein 1 (PD-1) and one of its ligands, programmed cell death 1 ligand 1 (PD-L1) on tumor cells and tumor infiltrating lymphocytes (TILs) to modulate T cell activity and suppress primary immune surveillance escape [4]. Studies of immune checkpoints, TILs, and tumor microenvironments (TME) are attracting increased attention. Immune profiling analysis of tumor tissue has become a vital part of immunotherapy studies, especially for discovering novel predictive biomarkers and detecting interaction characteristics between tumor cells and TILs, as well as among tumor associated immune cells (TAICs) [5].

Both basic and translational oncology research depend on experimental systems. The Mice model is an essential organism in cancer research, including cancer growth patterns, biological characteristics, the relationship between malignant cells and immune cells, and anti-cancer immune response to treatment, especially immunotherapy. Mice models like transplantable, genetically engineered, and carcinogen-induced malignancies have established the fundamental of cancer research [4].

As an essential technique for the research on tumor microenvironments, including TAICs and markers related to immunotherapy, multiplex immunofluorescence (mIF) can simultaneously detect multiple markers in a single formalin-fixed, paraffin-embedded (FFPE) tumor tissue, and make it possible for the evaluation of several effectors in a signal transduction pathway and distribution of molecules relative to each other within tumors at one time [6]. Compared to immunohistochemistry (IHC), which is widely used in clinical and basic research, there has been a limited number of mIF applied in mouse model cancer research, let alone clinical application. For the significant advantages of mIF analysis, promoting its application and development in animal cancer research is necessary.

We have established mouse panels for animal cancer research. In this study, our goal was to use this mIF panel and apply it in a small cohort of a lung adenocarcinoma mouse model to explore the versatility of detecting co-expression markers, especially the axis of PD-1/PD-L1, TILs and macrophages to see the ability of this data to explore the spatial distribution of different cell subtypes.

## Methods

### Tissue specimens and control tissue

Animal model was approved by Animal Care and Use Committee MD Anderson, Cancer Center. The approval number is ‘protocol RP160652’. LKR10/LKR10KO Kras-mutant murine lung adenocarcinoma cells (2 × 10^6^) were injected subcutaneously into the right flank of six syngeneic recipient male mice (129 Sv genetic background), similar to the previously protocol described [7]. After injection, tumor caliper measurements were performed twice a week. The mice were sacrificed when tumor volume reached 1,500 mm^3^ or when moribund. All mice were bred and housed under the same condition. The tissue samples were formalin-fixed paraffin-embedded. In parallel, two cases of mice lymph node FFPE tissues were obtained, ensuring they also contained adjacent glands for the cytokeratin staining. The thickness of tissue cutted from each FFPE block was 4 μm. Then the tissue was placed on charged positive slides, and prepared for uniplex and multiplex IF validation.

### Immunohistochemistry validation

Antibodies validated in mice panel contain cytokeratin 19 (CK19), CD3e, CD8a, CD4, PD-1, PD-L1, and F4-80. An automated staining system Leica BOND MAX (Leica Biosystems, Vista, CA) was used to perform chromogenic IHC analysis with antibodies against CK 19 (dilution, 1:20), CD3e (dilution, 1:100), CD8a (dilution, 1:200), CD4 (dilution, 1:100), PD-1 (dilution, 1:50), PD-L1 (dilution, 1:100) and F4-80 (dilution, 1:200 (Supplementary Fig. 1).

### IF antibodies and tyramide signal construction

Single immunofluorescence (IF) of all markers was performed automatically by using Leica Bond RX (Leica Biosystems, Vista, CA); Opal 7 kit (catalog #NEL797001KT; Akoya Biosciences, Marlborough, MA) tyramide sigfnal amplification (TSA) fluorophores, including 4′,6-diamidino-2-phenylindole (DAPI), Opal Polaris 480, Opal Polaris 520, Opal Polaris 540, Opal Polaris 570, Opal Polaris 620, Opal Polaris 650 and Opal Polaris 690, were used to combine with each antibody separately. Supplementary Table 1 shows the details of antibodies and fluorophores used.

### Processing of mIF

Hematoxylin (catalog #104,302, Merck, Germany) and eosin (catalog #109,844, Merck, Germany) (H&E) staining was performed before IF staining to employ tumor position and histomorphometric analyses. Uniplex IF staining of all the markers was performed using an automated staining system Leica Bond RX(Leica Biosystems, Vista, CA). Each antibody was linked by one fluorophore to detect the targets proposed in this mIF panel, optimize the antibodies, and build spectral libraries prepared for mIF image analysis. After baking and deparaffinization, firstly, slides were washed by Opal Detection Buffer, followed by two washes with Bond Wash Solution (1 × 2-methyl-2 H-isothiazol-3-one, catalog #AR9590, Leica Biosystems). Slides were washed and heated at 95℃ for 20 min by Bond Antigen Retrieval Tris-EDTA buffer or citrate buffer (depending on antibodies). After three times additional washes with Bond Wash Solution, slides were incubated with Immunofluorescence Blocking Buffer (Goat Serum Buffer for CK19 and Cell Signaling Technology, #12,411 for other targets) for 10 min at room temperature, followed by 30 min, 60 min, or 120 min incubation at room temperature with each antibody at a specific dilution (depending on antibodies). After incubation, the slides were washed three times with Bond Wash Solution. Used as a secondary antibody, polymer horseradish peroxidase (HRP for all except CK19) or rat HRP (for CK19) were incubated with slides for 10 min at room temperature. After five times successive washes with Bond Wash Solution, 10 min of incubation was performed on the slides with one of the TSA fluorophores corresponding to each antibody (according to the manufacturer’s instructions, correspondence, and dilution, shown in Supplementary Table 1). After four additional washes with Bond Wash Solution, the slides were washed and heated at 95℃ for 20 min by Bond Antigen Retrieval Tris-EDTA buffer or citrate buffer, and a new circle of the next antibody started. After the last process ended by four times wash with Bond Wash Solution, an additional two bond washes were performed. Then the slides were washed once and counterstained with DAPI for 6 min. After continuous flushing for 1 min, the slides were removed from the trays and manually mounted with ProLong Diamond Antifade Mountant (Invitrogen/Thermo Fisher Scientific, Waltham, MA). The sequence of antibodies in this panel was set up in the automated protocol (Supplementary Fig. 1) according to the request of obtaining a similar exposure time range from 50 to 150 ns of different antibodies conjugated with their fluorescent dyes [8].**Spectral library**.

The spectral library was created for the extraction of multispectral image visualization after assessing each target by a uniplex IF assay using the inForm image analysis software (InForm 2.4.8, Akoya Biosciences) (Supplementary Fig. 2). We set negative controls and created positive controls using different lymph nodes with the adjacent gland sections. Mouse lymph nodes were chosen as positive controls because they contain all the markers studied and their distribution pattern. After optimizing each antibody, they were combined for the mIF validation. PhenoImager HT 1.0.13 scanner system (Akoya Biosciences, Marlborough, MA) was used to scan stained slides and acquire fluorescence images.

### Image selection and analysis

The two lymph nodes with the adjacent glands used as positive controls were also scanned by the PhenoImager HT 1.0.13 scanner system (Akoya Biosciences, Marlborough, MA) to calibrate the spectral image protocol. Low magnification scanning of the slides was performed first, followed by random area selection of five individual fields (913 × 698 μm, 0.6372 mm^2^ each) in the intratumoral compartment with the selection software (Phenochart 1.0.12, Akoya Biosciences, Marlborough, MA). Then the selected regions would be scanned at 20× high resolution. The histologic assessment was performed using H&E staining slides to ensure that tumor tissue (at least 85% malignant cells, CK19 positive) was included in the selected intratumoral region. High-resolution images were assessed by InForm software, and a “spectral unmixing library” previously built was used to obtain spectral signatures for each fluorophore. The selected regions were divided into tumor-epithelial compartment (groups or nests of malignant cells) and tumor-stroma compartment (represented by the stroma area between tumor cells) according to the expression of CK19. The tumor cell and the immune cell populations from each image were identified and quantified using the cell segmentation and phenotype cell tool based on positive markers by the InForm image analysis software under pathologist supervision. Different cell phenotypes (Supplementary Fig. 3) were quantified, and the average of them was expressed in density per mm^2^. After completion of the analyses, data was sent to our data analyst to merge and consolidate each case using R studio 3.5.3 (Phenopter 0.2.2 packet; https://rdrr.io/github/akoyabio/phenoptrReports/f/, Akoya Biosciences, Marlborough, MA).

### Functional spatial analysis

To explore the phenotypic values within each compartment, we used spatial analysis to measure and evaluate the distance of each TAIC (T-cell phenotype CD3e^+^ and macrophage phenotype F4-80^+^) to malignant cells (CK19^+^). X and Y positions of each cell phenotype were used to calculate the nearest neighbor distances by R studio 3.5.3 (Phenopter 0.2.2 packet; https://rdrr.io/github/akoyabio/phenoptrReports/f/, Akoya Biosciences, Marlborough, MA), from CK19^+^ malignant cells to the different TAICs.

### Statistical methods

The densities and distances of various cell phenotypes from malignant cells were dichotomized: values greater than the median were considered high density or long distance, and values equal to or lower than the median was regarded as low density or close distance. Nonparametric tests were used to assess associations in the densities between compartments or spatial distance analysis from malignant cells to TAICs using the Wilcoxon rank-sum or Kruskal-Wallis test. An un-adjusted *P*-value of less than 0.05 was considered statistically significant. All analyses and data visualization were performed in R 3.6.0 (released April 2019; https://www.r-project.org), R studio 3.5.3 (Phenopter 0.2.2 packet; https://rdrr.io/github/akoyabio/phenoptrReports/f/, Akoya Biosciences, Marlborough, MA), and GraphPad Prism v.9.0.0.

## Results

### IHC validation and multiplex IF validation

Different markers were evaluated using chromogenic IHC approaches and uniplex IF, and obtained similar staining patterns in mice lymph node controls. As shown in Supplementary Fig. 1, cytokeratin 19 positive (epithelial cells in glands), CD3e positive (T lymphocytes), CD4 positive (helper T cells), CD8a positive (cytotoxic T cells), F4-80 positive (macrophages), PD-1 positive, and PD-L1 positive (membrane expression at any intensity) cells showed similar staining patterns and distribution with multiplex IF staining compared with IHC staining and uniplex staining in the mice lymph nodes controls, characterizing a successful optimization of the multiplex immunofluorescence panel.

### Immune cell phenotypes characterized in mice lung cancer

**Fig. 1:**
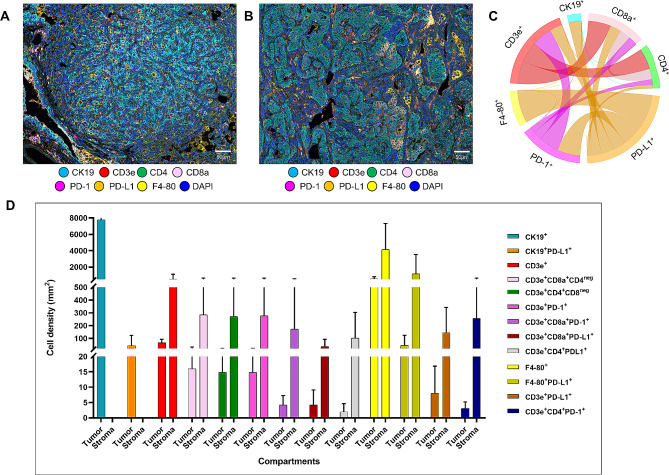
Representative examples of multispectral images, with chord diagram of the markers and densities of phenotypes. Composite spectral mixing images from multiplex immunofluorescence (mIF; 20× magnification, scale bars represent 50 μm on each image) is shown for (**A**-**B**) mouse panel: cytokeratin 19 (CK19), CD3e, CD4, CD8a, PD-1, PD-L1, and F4-80. (**C**) Chord diagram visualization showing the diversity of inter-relationships between markers’ co-expression in the mouse panel. (**D**) The images were generated using PhepoImager HT 1.0.13 scanner system and InForm 2.4.8 image analysis software (Akoya Biosciences). The chord diagram was generated by R studio software version 3.6.1. The graphic bar was generated using GraphPad Prism v.9.0.0

After getting satisfied mIF images of each marker in positive control tissues, we applied this panel in six mice lung adenocarcinoma specimens, as shown in representative examples in Fig. 1A-B, to explore TAIC populations. As demonstrated in the cord plot of Fig. 1C, we observed different interactions between markers in the panel. Different phenotypes of TAICs were identified using co-expression and co-localization of different markers (Supplementary Figs. 3 and 4). In this cohort, the phenotypes shown in the mice panel include malignant cells expressing PD-L1 (CK19^+^PD-L1^+^), cytotoxic T cells (CD3e^+^CD8a^+^CD4^neg^), helper T cells (CD3e^+^CD4^+^CD8a^neg^), antigen-experienced T cells (CD3e^+^PD-1^+^), antigen-experienced cytotoxic T cells (CD3e^+^CD8a^+^PD-1^+^), cytotoxic T cells expressing PD-L1+ (CD3e^+^CD8a^+^PD-L1^+^), antigen-experienced helper T cells (CD3e^+^CD4^+^PD-1^+^), helper T cells expressing PD-L1 (CD3e^+^CD4^+^PD-L1^+^), macrophage expressing PD-L1^+^ (F4-80^+^PD-L1^+^). We quantified all the cell subtypes and provided the cells’ density and/or percentage as well. All cell subtypes’ densities are shown in Tables 1 and Fig. 1D. Overall, the markers had similar median value densities. With a cutoff of greater than 1% of the malignant cells’ membrane expressing PD-L1, all six cases were classified as PD-L1 positives. However, we observed that in CK19^+^ malignant cells, only a median of 3.05 cell/mm^2^ (min 0.52 cell/mm^2^; max 200.09 cell/mm^2^) expressed PD-L1 (CK19^+^PD-L1^+^).

**Table 1:** Median cell densities by tumor compartments

Phenotype	Median cells densities (mm^2^) by tumor compartments			
	Total	*Tumor-epithelial	*Stroma	*P
CK19^+^	7403.26	7751.85	0.00	-
CK19^+^PD-L1^+^	3.05	3.08	0.00	-
CD3e^+^	75.66	61.08	248.49	0.200
CD3e^+^CD8a^+^CD4^neg^	16.56	12.40	116.07	0.078
CD3e^+^CD4^+^CD8a^neg^	19.05	16.20	84.51	0.336
CD3e^+^PD-1^+^	18.92	13.73	87.93	0.055
CD3e^+^PD-1^+^PD-L1^+^	7.35	3.37	59.28	1.000
CD3e^+^CD8a^+^PD-1^+^	5.00	3.59	22.23	1.000
CD3e^+^PD-L1^+^	7.98	4.59	64.52	0.336
CD3e^+^CD8a^+^PD-L1^+^	3.60	1.86	10.46	1.000
CD3e^+^CD8a^+^PD-1^+^PD-L1^+^	3.44	1.86	0.00	0.328
CD3e^+^CD4^+^PD-1^+^	4.92	3.14	40.29	0.336
CD3e^+^CD4^+^PD-L1^+^	2.73	1.23	18.17	0.618
CD3e^+^CD4^+^PD-1^+^PD-L1^+^	2.06	0.96	14.82	0.610
F4-80^+^	578.51	516.33	2966.41	0.004
F4-80^+^PD-L1^+^	27.72	16.52	130.38	0.630

*P, comparison between tumor epithelial and stroma compartments

Overall, all these six samples showed low densities of TAICs. As expected, the densities of TAICs were higher overall in the tumor-stroma compartment than in the tumor-epithelial compartment, as shown in Table 1. The Median of CD3e^+^ T cells was 75.66 cells/mm^2^ (min 27.16 cells/mm^2^; max 99.63 cells/mm^2^). The number of cytotoxic T cells (CD3e^+^CD8a^+^CD4^neg^; median, 57.67 cells/mm^2^; min 26.23 cells/mm^2^; max 175.35 cells/mm^2^) was the most significant subset among all CD3e^+^ T cell subsets. The median density of CD3e^+^CD4^+^CD8a^neg^ helper T cells was 19.05 cells/mm^2^ (min 3.10 cells/mm^2^; max 24.81 cells/mm^2^). We also observed antigen-experienced T cells (CD3e^+^PD-1^+^, median 18.92 cells/mm^2^, min 3.10 cells/mm^2^, max 25.44 cells/mm^2^) and PD-L1^+^ T cells (CD3e^+^PD-L1^+^, median 7.98 cell/mm^2^, min 1.03 cells/mm^2^, max 28.72 cells/mm^2^), demonstrating potential T cell-mediated suppressive axes within the TME. Other phenotypes observed were antigen-experienced cytotoxic T cells (CD3e^+^CD8a^+^PD-1^+^) and PD-L1^+^ cytotoxic T cells (CD3e^+^CD8a^+^PD-L1^+^). Although quantities of the T cell subtype were low, F4-80^+^ macrophages were abundant, with a median density of 578.51 cells/mm^2^ (min 279.97, max 996.36). F4-80^+^ macrophages expressing PD-L1 were also observed but showed a low density (median 27.72 cells/mm^2^, min 0.55 cells/mm^2^, max 270.14 cells/mm^2^).

### Exploratory functional spatial distribution

**Fig. 2:**
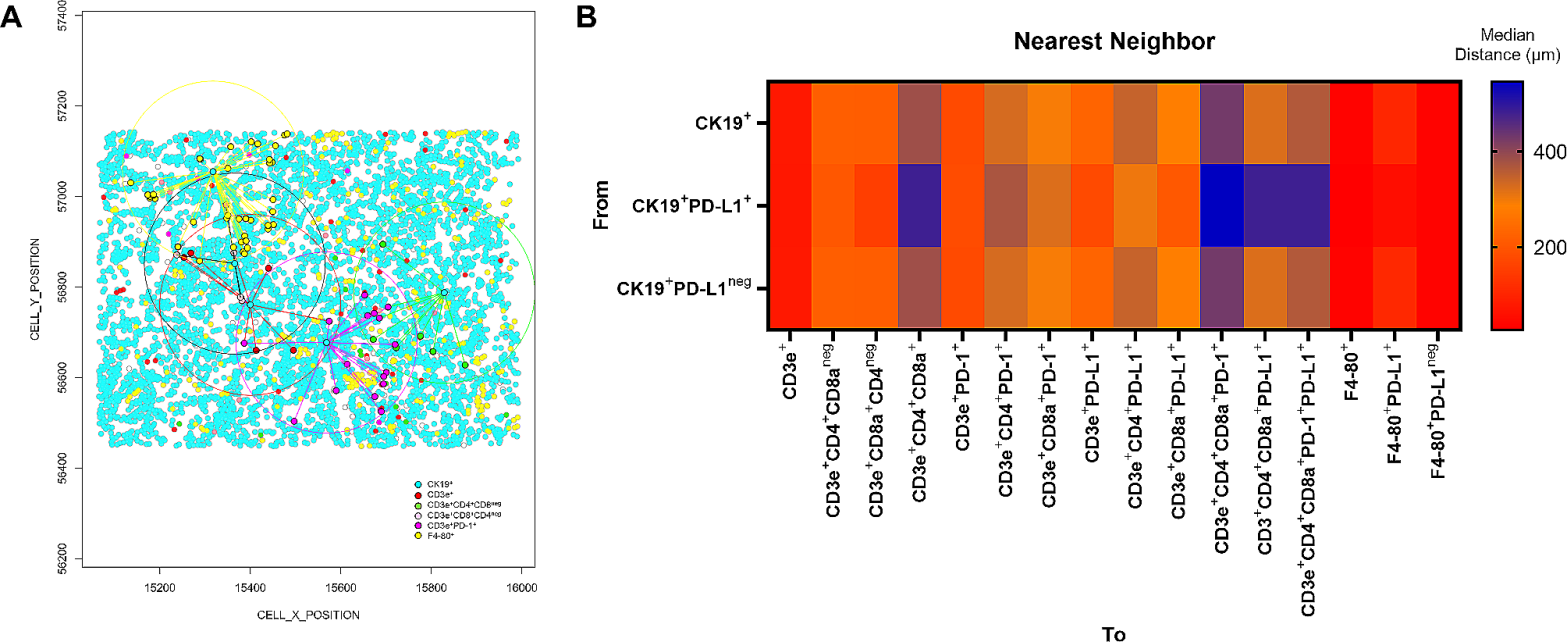
Representative graphic of neighbor distance and heat map representing distances from malignant cells to TAILs. (**A**) Representative example of neighbor distance calculation from malignant cells (CK19^+^, *cyan dotes*) to CD3e^+^ (*red dots*), CD3e^+^CD4^+^CD8^neg^ (*green dots*), CD3e^+^CD8a^+^CD4^neg^ (*pink dots*), CD3e^+^PD-1^+^ (*magenta dots*), and F4-80^+^ (*yellow dots*). (**B**) Median distance heat map representing different tumor-associated immune cells near or far from malignant cell phenotypes across the mouse cohort. Graphic bar was generated using R studio software version 3.6.1. Head map was generated using GraphPad Prism v.9.0.0

X and Y position of each cell was used to assess the nearest neighbor distances of TAICs from malignant cells. (Fig. 2A) We also created a matrix where each entry is the Euclidean distance from a pair of cells to evaluate the interaction between different cell phenotypes. This matrix identified the median distance of 229.30 microns from multiple TAIC phenotypes mentioned above to the malignant cells. We set the median distance as the overall radius distance. Based on this radius, the TAICs were separated into two groups, one with TAICs inside the radius and the other with TAICs outside the radius. In our cohort, the TAIC phenotypes closest to CK19^+^ malignant cells were F4-80^+^ macrophages, with a median distance of 31.44 microns (Table 2). Among these, most were PD-L1 negative macrophages. Total T cells (CD3e^+^), antigen-experienced T cells (CD3e^+^PD-1^+^), cytotoxic T cells (CD3e^+^CD8a^+^CD4^neg^) and helper T cells (CD3e^+^CD4^+^CD8a^neg^) were also observed close to malignant cells (median distance: 73.98, 175.40, 214.33 and 215.40 microns, respectively). We also observed that CD3e^+^CD4^+^CD8a^neg^ T cells, CD3e^+^CD4^+^CD8a^+^PD-1^+^ cells, and CD3e^+^CD4^+^CD8a^+^PD-1^+^PD-L1^+^ cells were far from malignant cells (median distance: 383.68, 427.73 and 363.90 microns, respectively). Median distance between malignant cells and CD3e^+^PD-L1^+^ T cells, CD3e^+^CD4^+^PD-1^+^ T cells, CD3e^+^CD4^+^PD-L1^+^ T cells, CD3e^+^CD8a^+^PD-L1^+^ T cells, CD3e^+^CD4^+^CD8a^+^PD-L1^+^ T cells was 227.63, 328.34, 344.54 and 321.83 microns, respectively.Interestingly, cytotoxic T cells (CD3e^+^CD8a^+^CD4^neg^), T cells expressing PD-L1 (CD3e^+^PD-L1^+^), macrophages (F4-80^+^), and macrophages expressing PD-L1 (F4-80^+^PD-L1^+^) were closer to PD-L1^+^ malignant cells (CK19^+^PD-L1^+^) than to PD-L1 negative malignant cells as shown by the head map in Fig. 2B. CD3e^+^ T cells with PD-1^+^ had a closer distance to both PD-L1^+^ malignant cells and PD-L1^neg^ malignant cells. (Table 2)

**Table 2:** Median distance in microns from malignant cell (CK19^+^) phenotype to tumor immune cells observed in the 6 mice lung adenocarcinoma cases

To phenotype	From CK19 phenotype		
	CK19^+^	CK19^+^PD-L1^+^	CK19^+^PD-L1^neg^
	Median distance in microns		
CD3e^+^	73.98	72.26	74.00
CD3e^+^CD4^+^CD8a^neg^	215.40	202.31	215.52
CD3e^+^CD8a^+^CD4^neg^	214.33	152.01	214.34
CD3e^+^CD4^+^CD8a^+^	383.68	481.60	383.41
CD3e^+^PD-1^+^	175.40	179.38	175.40
CD3e^+^CD4^+^PD-1^+^	328.34	371.52	328.37
CD3e^+^CD8a^+^PD-1^+^	292.98	321.48	292.87
CD3e^+^PD-L1^+^	227.63	176.05	227.61
CD3e^+^CD4^+^PD-L1^+^	344.54	302.35	344.00
CD3e^+^CD8a^+^PD-L1^+^	281.47	203.40	281.44
CD3e^+^CD4^+^CD8a^+^PD-1^+^	427.73	545.26	427.60
CD3e^+^CD4^+^CD8a^+^PD-L1^+^	321.83	481.60	321.81
CD3e^+^CD4^+^CD8a^+^PD-1^+^PD-L1^+^	363.90	481.60	363.86
F4-80^+^	31.44	26.94	31.44
F4-80^+^PD-L1^+^	103.25	53.21	105.94
F4-80^+^PD-L1^neg^	31.77	31.08	31.76

## Discussion

Till now, we have applied over ten multiplex immunofluorescences (mIF) panels for human tumors used in tumor research or clinical trials to study immune marker expression characteristics of tumor cells and TAICs before or after treatment, including chemotherapy and immunotherapy. Our mIF staining, scanning, and image analysis techniques were well-established and robust. The mIF proved to be an invaluable tool for tumor tissue immune profiling [8–10]. In this study, we established an mIF panel specially applied in mice tumors and explored the characteristics of TAICs in mice lung adenocarcinoma using the Opal workflow according to the rising research needs of mice tumors.

The Opal workflow of mIF used in this study allows simultaneous staining of eight biomarkers within a single FFPE tissue section. This panel could be applied in any type of mice carcinomas according to the markers included. After the antibodies and TSA fluorophores are manually prepared at specific dilution, they are put into the staining machine with different kinds of buffers, and all the following protocols are automatic. It takes about 17 h for the whole staining process automatically compared with three days manually, saving time, research reagents, and human resources. All the steps of our Opal workflow are detailed and precise, which allows accurate and reducible results. Images obtained from the PhenoImager HT scanning system could be reserved, which allows repeated analysis when getting a confusing result. According to our experience, diligent validation and optimization are required to obtain beautiful and satisfied staining of each target, first in IHC, then mIF in positive control tissue, and finally in tumor samples. We used to validate simplex IF after IHC validation and before the mIF of our first several human panels [6].

There are also other techniques on immune cell profiling, such as flow cytometry or multicolor flow cytometry, which could also identify cell subsets and is widely used in tumor immune profiling studies [11, 12]. But flow cytometers typically perform live cell measurements at one timepoint, and cannot be used in fixed tissue like FFPE tissue that is suitable for long-term storage. However, multiplex immunofluorescence could identify eight biomarkers within a single FFPE slide with high specificity and is reducible. No timepoint is limited when applying mIF on tumor tissue. In addition, our study could provide spatial information at single-cell level than flow cytometry. With spatial information, cell neighborhood analysis could be done and intercellular interactions could be predicted. Although there are only 6 cases in our cohort, specific features of TAICs could be explored and identified from each section. All mice were with similar physiological parameters, so we did not focus on the comparation of physiological parameters of individuals. In this study, we observed lower TAICs densities in mice lung adenocarcinoma than in previous studies of human lung adenocarcinoma [13–15]. Densities of CD3e^+^ T cells (total T cells) were also much lower in this cohort than in human lung cancer [13–17]. Interestingly, most TAICs subgroups showed significantly higher density in the tumor-stroma compartment than in the tumor-epithelial and epithelial-stroma compartments, as well as total TAICs, which is consistent with that in human lung cancer [18]. In human lung cancer, the density of CD3e^+^ T cells was the highest among all TAICs, and CD3e^+^ T cells in mice lung cancer had much lower density than in human lung cancer [17]. However, in our mice cohort, F4-80^+^ macrophages showed the highest density of all TAICs subgroups, even higher than the total number of all other subgroups. It suggests that macrophages play an essential role in modulating tumor immunity in mice lung cancer via macrophage-mediated T cell regulation or different pathways. Besides, the better air condition our mice model lived in and much shorter life time than human being might contribute to less lung inflammation, resulting in low density of T cell subtypes. We tried to compare with previous studies of mice lung cancer but did not find studies showing TAICs densities of mice lung cancer.

For spatial analysis, the first two TAIC subtypes closest to malignant cells were F4-80^+^ macrophages and CD3e^+^ T cells, which is similar to our previous finding in human lung cancer [17], suggesting these two subtypes played essential roles in lung cancer. The PD-L1 axis showed close interaction with TAICs. PD-L1^+^ malignant cells showed close proximity with cytotoxic T cells and macrophages, supporting that density and location played vital roles in lung cancer. Macrophages expressing PD-L1 and T cells expressing PD-L1 were closer to the malignant cells, suggesting that PD-L1 could guide specific cellular modulation between malignant cells and TAICs. Moreover, CD3e^+^PD-1^+^ experienced T cells showed close interaction with PD-L1^+^ malignant cells expressing.

There were several limitations associated with our study. Given the descriptive, exploratory nature of this study, the cohort was small, which is the main limitation of our study. Although this is a technical study and is limited to a specific mouse tumor using selected antibodies to study the axis of PD-1/PD-L1. Multiplex immunofluorescence is limited by the size of panels of fluorophore markers because of the overlaps in fluorescence-emission spectra. So, each panel contains up to 9 markers. The panel used in our study focused on T cells and PD-1/ PD-L1 axis. Myeloid-derived suppressor cells (MDSCs) are critical components of the tumor microenvironment (TME). Markers of MDSCs include CD11b, CD14, CD16, Arginase 1 (Arg 1), HLA-DR, CD33 and CD163. It is also necessary to develop panels containing MDSCs markers as well as other markers like CD19 (B cells), Granzyme B (activated T cells), Foxp3 (regulatory T cells), α-SMA (cancer associated fibroblasts, CAFs) and FAP (CAFs) to characterize the TME. This technology can be applied to different tumor types, changing the tumor marker target and a large quantity of data could be obtained. Our findings will be extended into more tumors and applied to more studies.

In summary, we demonstrated that this mIF mouse panel staining, targeting different antibodies in the same tissue section, gives us high-quality data. The immune-suppressive microenvironment observed in our small cohort driven by PD-L1/PD-1 axis suggests that this panel can help to profile lung adenocarcinoma in mouse models. Comprehensive immuno-profiling using mIF panels will improve our understanding of how different factors can determine disease progression, resistance, or response to immunotherapies and can help determine new treatment approaches using mouse models. Further studies in large cohorts of muse tissue may help to answer several questions and validate the use of this type of mIF panel to study cancer in mouse models, especially cancers treated with immunotherapy.

## Conclusions

We successfully created an mIF panel for mice lung adenocarcinoma, estimating important immune markers and tumor immune profiling with tyramide signal amplification technology. This would be applied to translational research and provide a new method of animal tumor research. TAICs analysis by mIF allows quantitative, automated staining, provides cell densities of specific phenotypes, and shows spatial interaction among immune and malignant cells in different tumor compartments. As far as we know, our study is the first one exploring the PD-1/PDL1 axis in mice tumors using mIF technology.

### Electronic supplementary material

Below is the link to the electronic supplementary material.

Supplementary Material 1: Fig. 1. Representative examples of multispectral unmixed, mixed images and immunohistochemistry from the different marker. Unmixed fluorescence markers of CK19, CD3e, CD4, CD8a, PD-1, PD-L1, and F4-80 plus their corresponding chromogenic immunohistochemistry and composite spectral mixing image from the multiplex immunofluorescence panel in lymph node mouse tissue (IF, IHC, and mIF; 20× magnification, scale bars represent 50 μm on each image). mIF images were generated using PhenoImager HT 1.0.13 scanner system and InForm 2.4.8 image analysis software (Akoya Biosciences).

Supplementary Material 2: Fig. 2. Generation of the spectral library for the fluorescence image scanning calibration. The spectral library showing the picks of the fluorescence wailing from each specific fluorophore used in the mouse panel.

Supplementary Material 3: Fig. 3. Representative examples of co-expression images from the mouse multiplex immunofluorescence panel. Unmixed fluorescence markers of CK19, CD3e, CD4, CD8a, PD-1, PD-L1, and F4-80 and their co-expression in a composite spectral mixing image were observed across the lung adenocarcinoma mouse model. IF and mIF images were generated using PhenoImager HT 1.0.13 scanner system and InForm 2.4.8 image analysis software (Akoya Biosciences).

Supplementary Material 4: Fig. 4. Representative examples of multispectral unmixed and mixed images analyzed from the different marker. Unmixed fluorescence markers of CK19, CD3e, CD4, CD8a, PD-1, PD-L1, and F4-80 and its mixed compose image. IF and mIF images were generated using PhenoImager HT 1.0.13 scanner system and InForm 2.4.8 image analysis software (Akoya Biosciences).

Supplementary Material 5: Table 1. Antibody optimization by multiplex immunofluorescence using the fluorophores contained in the Opal 7 kit and the fluorophore 480 (Akoya Biosciences)

## Data Availability

The authors declare that the data supporting the findings of this study are available within the manuscript and its supplementary information files. The data is provided as a source data file. Other data related to the current study are available in the repository of MD Anderson Cancer Center from the corresponding author (E.R.P) upon academic request to sign a data access agreement with the University of Texas MD Anderson Cancer Center after approval.

## Abbreviations

CK19: cytokeratin 19
FFPE: formalin-fixed, paraffin-embedded
IHC: immunohistochemistry
mIF: multiplex immunofluorescence
NSCLC: non-small cell lung cancer
PD-1: programmed cell death protein 1
PD-L1: programmed cell death 1 ligand 1
TAICs: tumor associate immune cells
TILs: tumor-infiltrating lymphocytes
TME: tumor microenvironmentTSA tyramide signal amplification
