# Supplementary figures and images for: Immune profiling of mouse lung adenocarcinoma paraffin tissues using multiplex immunofluorescence panel: a pilot study
Source: Lab Anim Res. 2024 Jun 14;40:24. doi: 10.1186/s42826-024-00210-w (PMC11177412; doi:10.1186/s42826-024-00210-w)

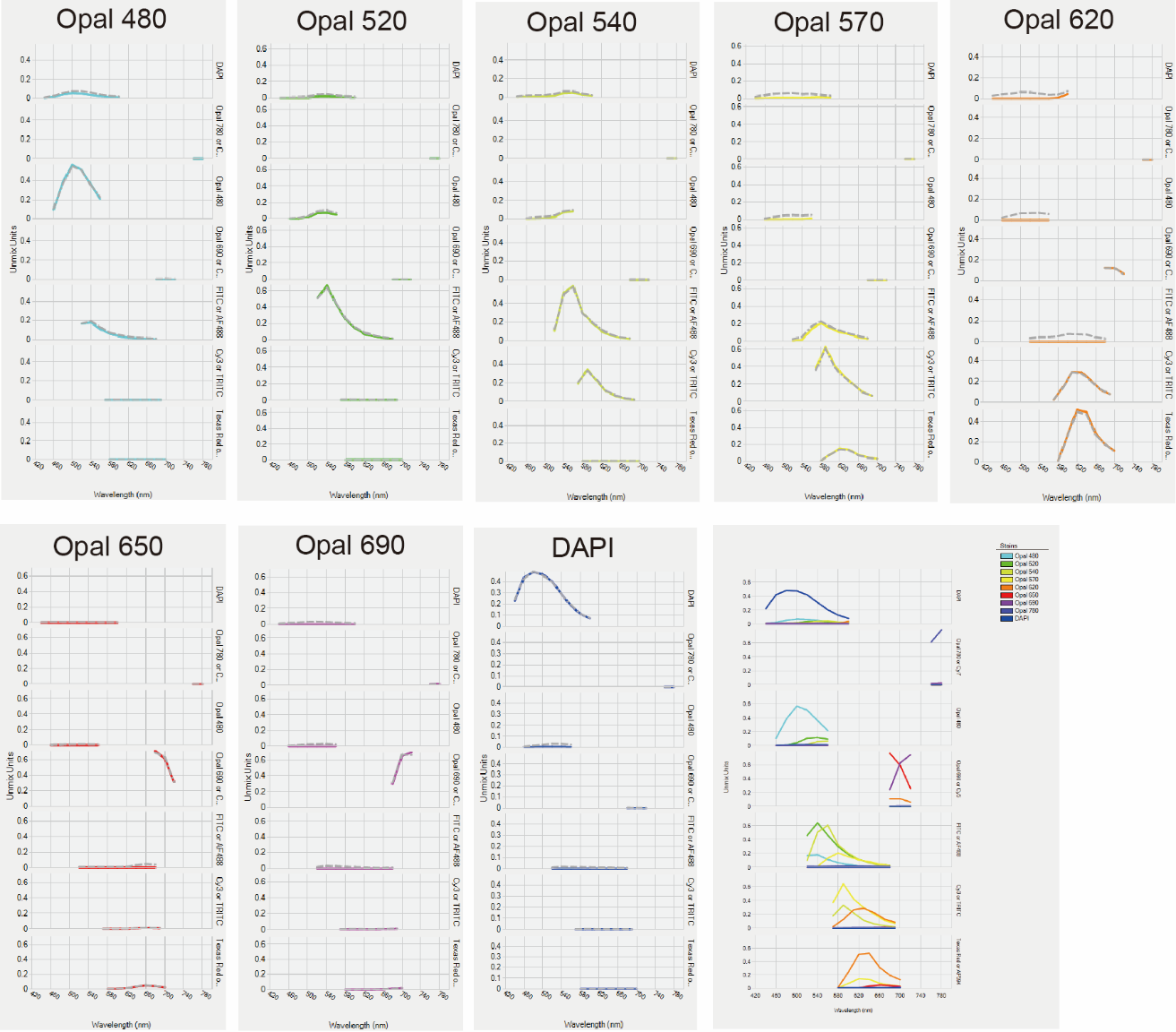

Supplement: Supplementary file 1 — Supplementary Material 1: Fig. 1. Representative examples of multispectral unmixed, mixed images and immunohistochemistry from the different marker. Unmixed fluorescence markers of CK19, CD3e, CD4, CD8a, PD-1, PD-L1, and F4-80 plus their corresponding chromogenic immunohistochemistry and composite spectral mixing image from the multiplex immunofluorescence panel in lymph node mouse tissue (IF, IHC, and mIF; 20× magnification, scale bars represent 50 μm on each image). mIF images were generated using PhenoImager HT 1.0.13 scanner system and InForm 2.4.8 image analysis software (Akoya Biosciences). [file 42826_2024_210_MOESM1_ESM.docx]

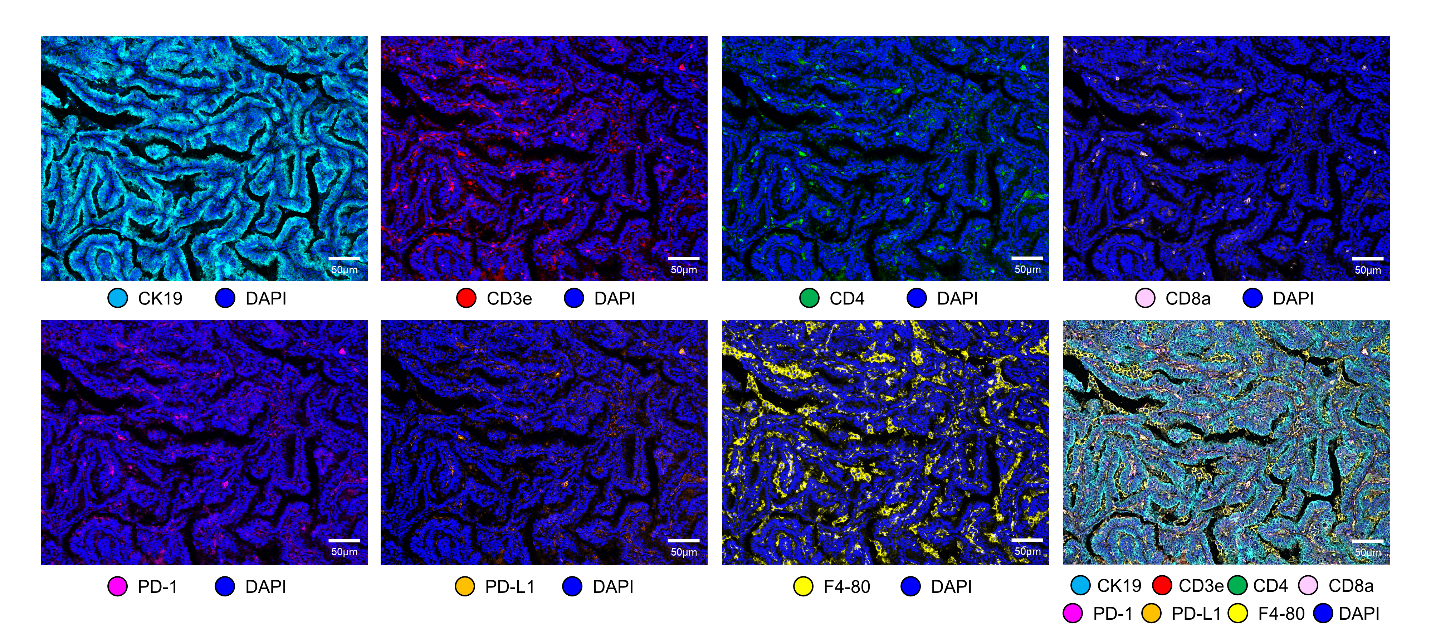

Supplement: Supplementary file 2 — Supplementary Material 2: Fig. 2. Generation of the spectral library for the fluorescence image scanning calibration. The spectral library showing the picks of the fluorescence wailing from each specific fluorophore used in the mouse panel. [file 42826_2024_210_MOESM2_ESM.docx]

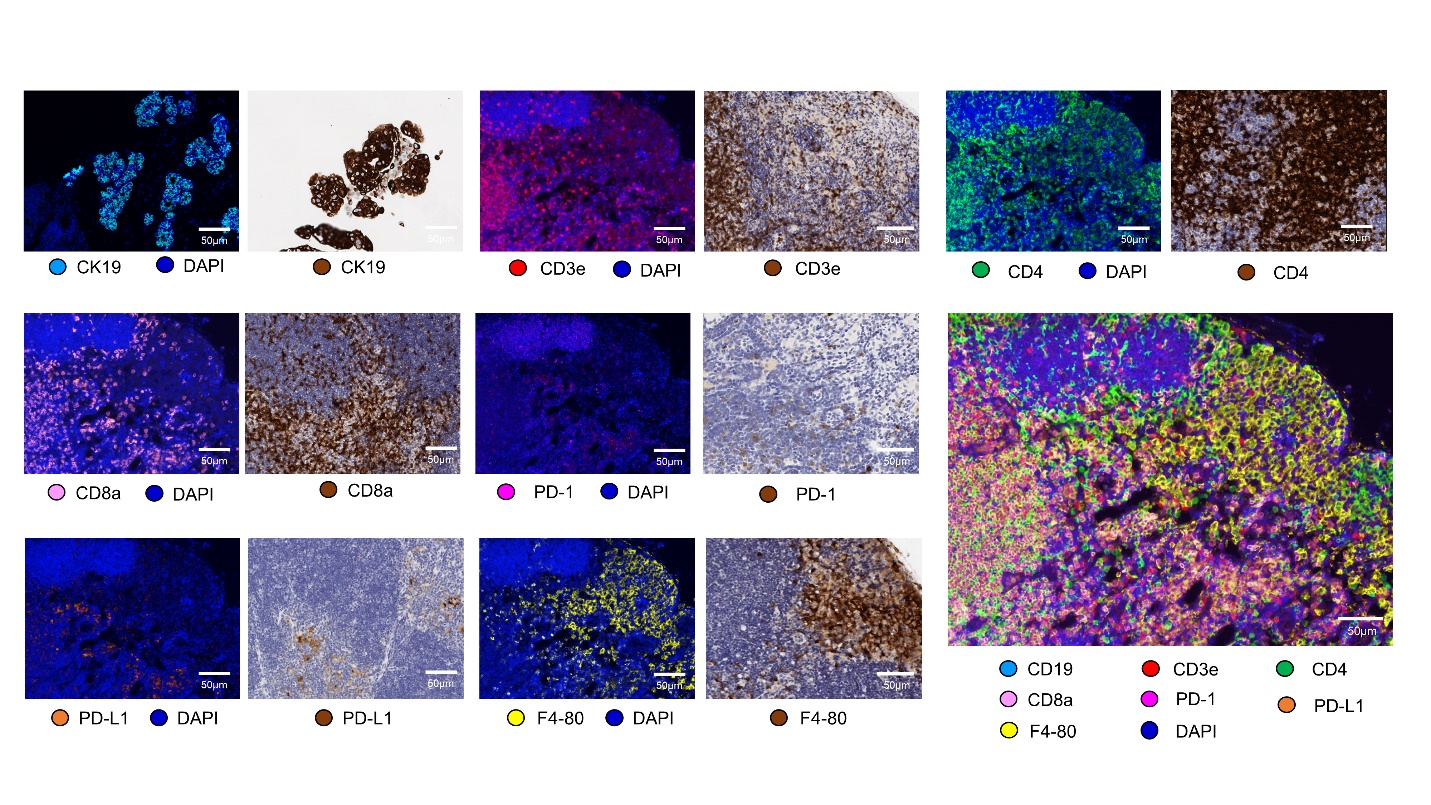

Supplement: Supplementary file 3 — Supplementary Material 3: Fig. 3. Representative examples of co-expression images from the mouse multiplex immunofluorescence panel. Unmixed fluorescence markers of CK19, CD3e, CD4, CD8a, PD-1, PD-L1, and F4-80 and their co-expression in a composite spectral mixing image were observed across the lung adenocarcinoma mouse model. IF and mIF images were generated using PhenoImager HT 1.0.13 scanner system and InForm 2.4.8 image analysis software (Akoya Biosciences). [file 42826_2024_210_MOESM3_ESM.docx]

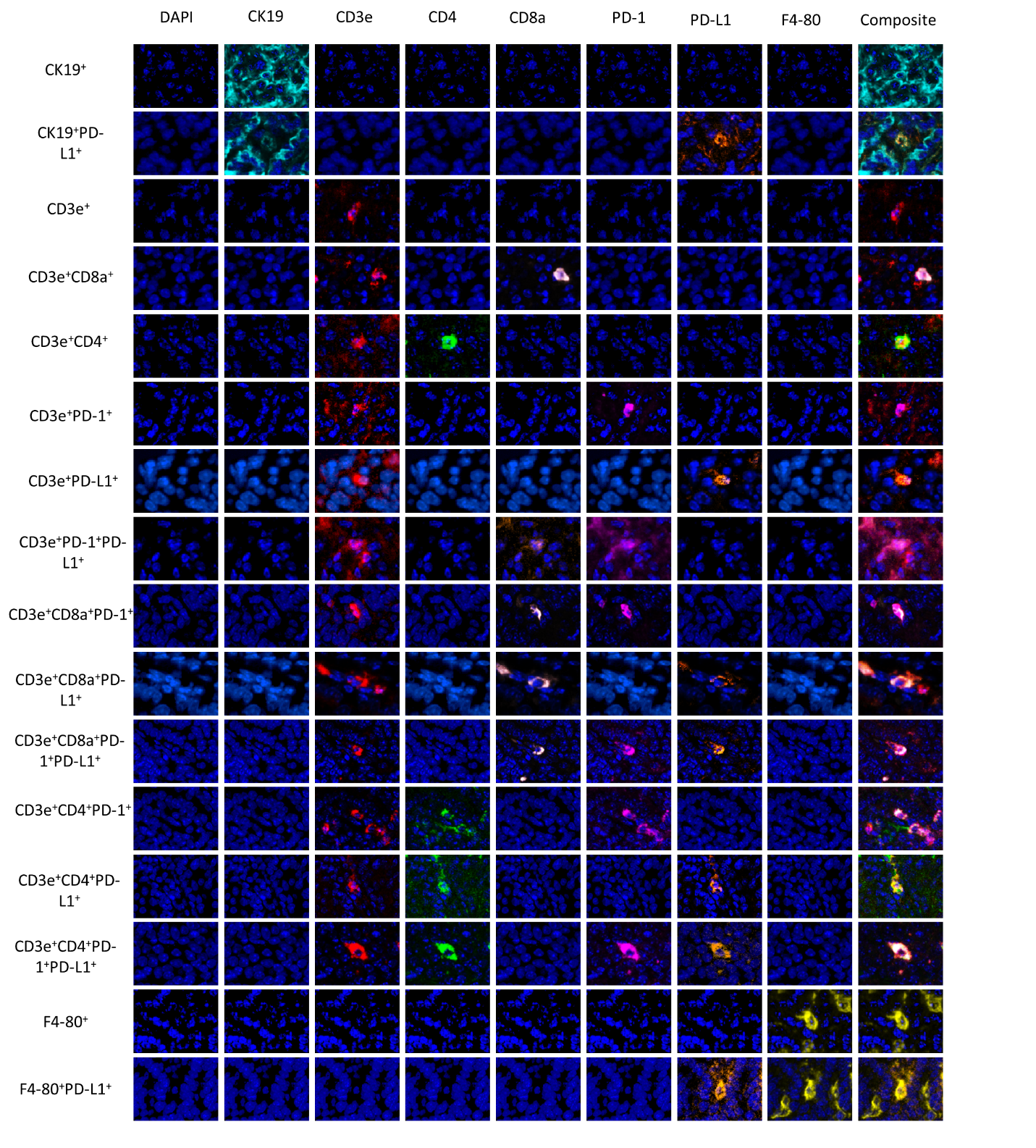

Supplement: Supplementary file 4 — Supplementary Material 4: Fig. 4. Representative examples of multispectral unmixed and mixed images analyzed from the different marker. Unmixed fluorescence markers of CK19, CD3e, CD4, CD8a, PD-1, PD-L1, and F4-80 and its mixed compose image. IF and mIF images were generated using PhenoImager HT 1.0.13 scanner system and InForm 2.4.8 image analysis software (Akoya Biosciences). [file 42826_2024_210_MOESM4_ESM.docx]
