# Supplementary material for: Immune profiling of mouse lung adenocarcinoma paraffin tissues using multiplex immunofluorescence panel: a pilot study
Source: Lab Anim Res. 2024 Jun 14;40:24. doi: 10.1186/s42826-024-00210-w (PMC11177412; doi:10.1186/s42826-024-00210-w)
Supplement: Supplementary file 5 — Supplementary Material 5: Table 1. Antibody optimization by multiplex immunofluorescence using the fluorophores contained in the Opal 7 kit and the fluorophore 480 (Akoya Biosciences) [file 42826_2024_210_MOESM5_ESM.docx]

**Supplementary Table 1.** Antibody optimization by multiplex immunofluorescence using the fluorophores contained in the Opal 7 kit and the fluorophore 480 (Akoya Biosciences)

| **Antibody** | **Clone** | **Vendor** | **Catalog #** | **AR** | **Ab. Dilution** | **F. Dilution** | **F** |
| --- | --- | --- | --- | --- | --- | --- | --- |
| CK 19 | TROMA-III | DSHB | NA | Ph9 | 1:20 | 1:100 | 480 |
| CD3e | D4V8L | CST | 99940S | Ph9 | 1:100 | 1:100 | 690 |
| CD4 | D7D2Z | CST | 25229S | Ph9 | 1:100 | 1:100 | 520 |
| CD8a | D4W2Z | CST | 98941S | Ph9 | 1:200 | 1:100 | 540 |
| PD-1 | D7D5W | CST | 84651S | Ph9 | 1:50 | 1:100 | 650 |
| PD-L1 | D5V3B | CST | 84651S | Ph9 | 1:100 | 1:100 | 570 |
| F4-80 | D2S9R | CST | 70076S | Ph6 | 1:200 | 1:100 | 620 |

AR: Antigen retrieval; Ab: Antibody; F: Fluorophores.
